# Delays to treatment initiation is associated with tuberculosis treatment outcomes among patients on directly observed treatment short course in Southwest Ethiopia: a follow-up study

**DOI:** 10.1186/s12890-018-0628-2

**Published:** 2018-05-02

**Authors:** Abyot Asres, Degu Jerene, Wakgari Deressa

**Affiliations:** 1 Department of Public Health, College of Health Sciences, Mizan Tepi University, Mizan Aman, Ethiopia; 2 Management Science for Health, Addis Ababa, Ethiopia; 3 Department of Preventive Medicine, School of Public Health, College of Health Sciences, Addis Ababa University, Addis Ababa, Ethiopia

**Keywords:** TB, Delay, Follow-up, Log-binomial, Ethiopia

## Abstract

**Background:**

Despite reported long delays to initiate anti-TB treatment and poor outcomes in different parts of Ethiopia and elsewhere, evidences on association between the delay and treatment outcomes are scanty.

**Methods:**

A follow up study among 735 new TB cases registered at health facilities in districts of southwest Ethiopia was conducted from January 2015 to June 2016. Patients reported days elapsed between onset of illness and treatment commencement of 30 days cutoff was considered to ascertain exposure. Thus, those elapsed beyond 30 days to initiate anti-TB treatment since onset of illness were exposed and otherwise non-exposed. The cases were followed until earliest outcome was observed. Treatment outcomes was ascertained as per the World Health Organization standard definitions and dichotomized into ‘successful’ when cured or treatment completed and ‘unsuccessful’ when lost to follow-up or died or treatment failure. Bivariate and multiple log-binomial models were fitted to identify predictors of unsuccessful outcomes.

**Results:**

The overall treatment success among the treatment cohort was 89.7% (88.4% vs. 94.2%, *p* = 0.01 respectively among those initiated treatment beyond and within of 30 days of onset of illness. Higher risk of unsuccessful outcome was predicted by treatment initiation beyond 30 days of onset [Adjusted Relative Risk (ARR) = 1.92, 95%CI:1.30, 2.81], HIV co-infection (ARR = 2.18, 95%CI:1.47, 3.25) and received treatment at hospital (ARR = 3.73, 95%CI:2.23, 6.25). On the other hand, lower risk of unsuccessful outcome was predicted by weight gain (ARR = 0.40, 95%CI:0.19, 0.83) and sputum smear negative conversion (ARR = 0.17,95% CI:0.09, 0.33) at the end of second month treatment.

**Conclusion:**

Higher risk of unsuccessful outcome is associated with prolonged days elapsed between onset of illness and treatment commencement. Hence, promotion of early care seeking, improving diagnostic and case holding efficiencies of health facilities and TB/HIV collaborative interventions can reduce risk of unsuccessful outcome.

**Electronic supplementary material:**

The online version of this article (10.1186/s12890-018-0628-2) contains supplementary material, which is available to authorized users.

## Background

Despite the fact that nearly all cases can be cured, tuberculosis (TB) remained to be among the major global public health problems. Globally, 10.4 million incident cases, of whom 6.1million notified and 1.4 million deaths were estimated to occur in 2015 [1]. Thus, the latest strategy, “End TB” has been designed in line with the sustainable development goals (SDG). The strategy aimed at reaching 90% case detection and treatment success by 2025 [2].

Tuberculosis has been recognized as a major public health problem in Ethiopia and efforts to control the disease has begun since early 1960s [3]. Nonetheless, TB has remained among the major public health problems in the country [4]. Accordingly, Ethiopia has been listed among the 14 TB, TB/HIV (Human Immunodeficiency Virus) and Multi-Drug Resistant TB (MDR TB) High Burden Countries (HBC) [1]. The first national TB prevalence survey conducted in 2011 revealed smear positive pulmonary TB(PTB) prevalence of 108/100,000; of which 55% were not detected before the survey [5] . In 2015, about 205,463 new cases [6] and 29,000 deaths were estimated to occur in the country [1] when only 125,801 (61%) were notified to National TB control Program (NTP) [6].

The ultimate goal of any TB control program is reduction of morbidity and mortality among patients and stop transmission through curing infectious cases [7]. Hence early detection and treatment of cases have been a pillar for the end TB strategy [2]. Nonetheless, studies from different parts of Ethiopia reported patients elapse too long time to initiate care seeking and treatment. TB patients elapse median of 30 [8, 9] in Northern Ethiopia and 63 days in Bale [10] to initiate care seeking and median of six in Addis Ababa [11] and 34 days in Bale [10] to commence treatment.

Delays to diagnosis and treatment of TB result in more serious illness, increased length of infectiousness and poor treatment outcomes including mortality and drug resistance [12–16]. It is also reported that the high mortality rate among people living with HIV is also partly explained by delays to TB treatment [16, 17]. In contrast, a study in Tel Aviv reported no association between treatment success and delay in treatment initiation [18].

Achieving high cure rates help to reduce transmission of TB and attract the great majority of existing cases to seek treatment [19]. As a result, treatment of TB is beyond treating an individual patient, rather it is considered as a public health intervention. Globally, 83% of cases successfully completed their treatment in 2015,and effective diagnosis and treatment of TB saved an estimated 49 million lives between 2000 through 2015 [1]. In Ethiopia, 89% (range: 69%–95%)of treatment cohort in different regions attained successful TB treatment [6]. However, cure rates of 81% (range: 38% to 92%) [4] across regions of the country have been reported.

Studies uncovered that patient demographics, clinical, bacteriologic and HIV co morbidity were associated with unsuccessful TB treatment outcomes [20–23]. Despite the prevailing long delays to initiate anti-TB treatment and poor outcomes in different parts of Ethiopia and elsewhere, evidences on effect of the delay on treatment outcomes are scanty. Hence, this study assessed effect of delayed treatment initiation on outcomes of TB. Evidences on the association between treatment delay and outcome is crucial to realize the targets of ending TB epidemic [2].

## Methods

### Study setting

The study was conducted in 14 health facilities (three hospitals and 11 health centers) in three zones of Southern Nation, Nationalities, and Peoples Region (SNNPR) of Ethiopia. The SNNPR is one of the nine Regional States in the country with an estimated 15.7million population in 2017 [24]. The three study Zones (Bench Maji, Kaffa and Sheka) are located at the southwestern part of Ethiopia bordering South Sudan and harbor an estimated 1.8million peoples [24]. The zones (an administrative unit that liaison *woredas* with the region) are organized into four town administrations and 26 *woredas*(administrative unit equivalent to districts). At the time of the study, three hospitals and 65 health centers were providing TB Directly Observed Treatment Short course (DOTS) services. However, all the hospitals and only 25 health centers were providing TB/HIV collaborative interventions [25].

Diagnosis and treatment of all forms of TB across Ethiopia has been based on national TB control guidelines that specify case definitions, diagnostic and treatment standards [26]. Thus all TB cases enrolled for this study were diagnosed using either sputum smear microcopy or clinical signs aided with x-ray. Those cases with sputum smear positive were labeled as smear positive pulmonary TB and negatives were smear negative pulmonary TB. Diagnosis of smear negative and extra-pulmonary TB were made using diagnostic algorithm recommended by the national guideline [26]. Detail definitions for each cases presented in definition of terms section. Previously, TB diagnosis and treatment had been limited to public hospitals and health centers, but public private mix (PPM) DOTS and community DOTS to public health posts have been recently introduced. Thus, in 2011, 92% of public hospitals and 95% of health centers, 2100 health posts and 317 PPM–DOTS centers were providing DOTS based diagnosis and/or treatment of TB in the country [27].

### Study design and sampling

A prospective cohort study among new TB cases was carried out from January 2015 through June 2016. The treatment cohorts were recruited from baseline survey designed to determine delays to initiate treatment. Since no standard cutoff point for delay to treatment, a clinical sound cutoff of 30 days was used to ascertain exposure. Thus TB cases who elapsed beyond 30 days to initiate treatment since onset of illness were labeled as exposed and those started treatment within 30 days of onset of illness were non exposed. Then the cases were followed until earliest treatment outcome. The sample size was computed using StatCalc program of EpiInfo at 95% significance level, 80% power, expected outcome (unsuccessful of 3% [15] among those delayed beyond 30 days) to detect a difference of 7% with those initiated treatment earlier and considering design effect of 1.5 and 10% lost to follow up a total of 640 cases were required., However, the sample size for assessing predictors of delaycomputed using the same procedure provided a total of 802 new cases . Hence, the same cases were followed for the outcome; the larger sample of 802 was taken the final sample size.

The sample was proportionally allocated to the zones, *woredas* and health facilities based on TB cases reported during the year preceding the study. Finally, consecutive consenting cases from were prospectively enrolled until the required sample was reached and followed until completion of the six-month treatment or earliest outcome. During the enrollment those new cases, older than 18 years of age, on intensive phase treatment were included and those transferred out and died before the interview were excluded from the study.

### Data collection

A structured questionnaire adapted from tools used elsewhere [5, 28] and standard TB register book [26] was used to gather the data. Besides, data abstraction checklist was prepared to draw clinical, bacteriologic and treatment outcomes of the patients from TB register. The questionnaire was translated into national language (*Amharic*) spoken by almost all residents in the study area. Ten diploma graduate nurse data collectors and three public health specialist supervisors were recruited and trained for 3 days. The training included description of questionnaire, interviewing techniques and pretest among TB cases on DOTS at nearby health facilities not included in the study. Finally, eligible cases were traced from the unit TB register and interviewed for sociodemographic, health care seeking, and treatment practices.

### Exposure ascertainment

The main exposure variable was delays to treatment measured by days elapsed between onsets of illness to initiation of anti-TB treatment (total delay). The total delay comprises both patient and provider delays. Patient delay was assessed by asking patients to recall and estimate date or number of days elapsed between onset of TB constitutional symptoms such as cough, fever, night sweats, chest pain, weight loss and loss of appetite until formal care seeking. Similarly, provider delay was estimated by asking date or number of days elapsed between first formal health care facility visits to anti-TB treatment initiation. Finally, total delay was computed as a sum of patient and provider delay or number of days elapsed between onsets of illness to initiation of anti-TB treatment. Since no standard cut off for delay, a clinically sound cut off of 30 days delay was taken to define exposure status. Therefore, cases who delayed beyond 30 days were categorized as exposed and those initiated within 30 days were grouped as non-exposed.

### Outcome ascertainment

The main outcome variable was treatment outcome which was ascertained based on standard definitions [26, 29]. Accordingly, outcomes were categorized into successful when the TB patient completed treatment with or without evidence of cure and unsuccessful when died or lost to follow-up or treatment failure. So that, coding was made as unsuccessful outcomes = 1 and successful = 0.

### Data processing

Data were entered in to Epi-Data V 3.5 and processed on SPSS version 21 and/or STATA version 13. The data were described using frequencies, proportions, mean, median, inter-quartile range and standard deviation as appropriate. Both the baseline and follow-up data were described and compared across the exposed and non-exposed groups. Categorical variables were compared using chi square test and numeric variables using independent and paired t tests as appropriate.

Information provision adequacy during treatment initiation was assessed based on 15 items constructed from TB treatment guidelines. The internal consistency of the items was checked by Cronbach’s Alpha (α) =0.87). A score of one is given for proper information and zero otherwise. So that information adequacy index was computed and labeled adequate when above median and inadequate when below median scores.

Association between the exposure (exposed vs, non-exposed) and outcome (unsuccessful vs. successful) was determined by log-binomial regression. Accordingly, bivariate and multiple log-binomial regression models were fitted to estimate crude and adjusted relative risk (RR) of unsuccessful outcome. In all the statistical tests, significance was judged at *p* value < 0.05.

### Ethical issues

The study was ethically approved by the Institutional Review Board (IRB) of college of Health Sciences at Addis Ababa University. Informed written consent was sought from patients before the two interviews, during the intensive phase and end of treatment.

### Definition of terms

- New case those never been treated for TB or have taken anti-TB drugs for less than 1 month.
- Smear positive PTB is a patient with at least two sputum smear examinations positive for Acid Fast Bacilli (AFB) by direct microscopy.
- Smear negative pulmonary TB is a patient having symptoms suggestive of TB with at least three initial smear examinations negative for AFB by direct microscopy, and no response to a course of broad-spectrum antibiotics, and again three negative smear examinations by direct microscopy and radiological abnormalities consistent with pulmonary tuberculosis, and decision by a clinician to treat with a full course of anti- tuberculosis
- Extra pulmonary TB is TB in organs other than the lungs, proven by histo-pathological evidence from a biopsy, Or based on strong clinical evidence consistent with active EPTB and the decision by a physician to treat with a full course of anti-TB therapy.
- Cured: A bacteriologically confirmed pulmonary TB case at the beginning of treatment who was smear or culture-negative at last month of treatment and on at least one previous occasion.
- Treatment completed: A TB patient who completed treatment without evidence of failure BUT with no record to show that sputum smear or culture results in the last month of treatment and on at least one previous occasion were negative
- Treatment failure: TB patient whose sputum smear or culture is positive at month 5 or later during treatment.
- Died: TB patient who dies for any reason during the course of treatment.
- Lost to follow up: TB patient interrupted treatment for 2 or more consecutive months.
- Treatment outcome not evaluated TB patient for whom no treatment outcome is assigned including cases “transferred out” to another treatment unit.

## Results

### Profile of study participants

**Fig. 1:**
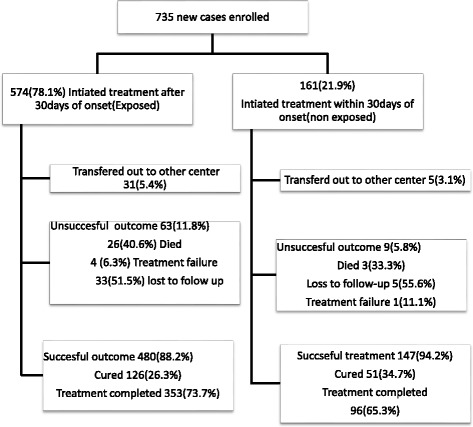
Flow chart of exposure and outcome ascertainment of TB cases Southwest Ethiopia,January 2015 to June 2016

A total of 735 (91.6%) of TB cases required were enrolled from 11 health centers and 3 hospitals. The rest of cases 67(8.4%) were not retrieved due to refusals (9), inability to respond (3) and end of the survey time (55). Of the cases 574 (78.4%) and 161(21.9%) had initiated anti-TB treatment within and above 30 days of onset of illness, respectively. Of the cases enrolled, 699 (95.1%) had documented treatment outcome and analyzed (Fig. 1).

### Baseline sociodemographic characteristics of study participants

The median age [inter-quartile range (IQR)] and of the cases was 27(20–37) years. Among the cases, 52.9% and 29.4% had completed elementary school and involved in subsistence farming respectively (Table 1).

**Table 1:** Socidemographic characteristics and delays to treatment in southwest Ethiopia January to December 2015

Variable		Total delay(days)		*P* value	Total N(%)
		> 30 (Exposed) *N* = 574	<=30 (Non exposed) *N* = 161		
		n(%)	n(%)		
Gender	Male	345(60.1)	102(63.4)	0.6	447(60.8)
	Female	229(39.9)	59(36.6)		288(39.2)
Age group (years)	18–34	389(67.8)	114(70.8)	0.04	503(68.4)
	35–65	173(30.1)	43(26.7)		216(29.4)
	> 65	12(2.1)	4(2.5)		16(2.2)
Residence	Urban	280(48.9)	88(54.7)	0.7	368(50.1)
	Rural	294(51.1)	73(45.3)		367(49.9)
Marital status	Never married	208(36.2)	67(41.6)	0.3	275(37.4)
	Currently married	315(54.8)	89(55.3)		404(55.0)
	Divorced/widowed	51(9)	5(3.1)		56(7.6)
Educational status	Illiterate	168(29.3)	44(27.3)	0.2	212(28.8)
	Completed primary	309(53.8)	80(49.7)		389(52.9)
	Secondary & above	97(16.9)	37(23.0)		134(18.2)
Occupation	Employed	134(23.3)	38(23.6)	0.01	172(23.4)
	Farming	163(28.4)	53(32.9)		216(29.4)
	Unskilled work ^a^	42(7.3)	9(5.6)		51(6.9)
	Dependents ^b^	235(40.9)	61(37.9)		296(40.3)

^a^housemaid, daily laborer, ^b^students, housewife

### Care seeking and treatment practices

Patients initially presented to healthcare facility after a median (IQR) 25(15–36) days (patient delay) since the onset of illness. Diagnosis of 623(84.7%) of the cases were made after an average (±SD) of 3.6(±2.4) visits to an average (±SD) of 2.2(±1.2) healthcare facilities at which time the patients had been treated with different medicines. The rest of the cases, 112(15.3%) were diagnosed at their first visit to the first health facilities. Thus diagnosis and initiation of anti-TB treatment took a median (IQR) of 22(9–48) days (provider delay) since the first visit to health care facilities. Generally, a median (IQR) 55(32–100) days (total delay) had been elapsed to initiate treatment since the onset of illness. Of the total cases, 373(50.7% were smear positive pulmonary in whom diagnosis was bacteriologicaly confirmed using sputum smear microscopy. All of the cases were offered HIV screening test, of whom 68 (9.3%) tested positive (95% CI: 7.2%–11.3%)). Of those TB/HIV co infected cases, 27(39.7%) and 32(47.1%) were respectively receiving antiretroviral therapy (ART) and Cotrimoxazole Prophylactic Therapy (CPT)(Table 2).

**Table 2:** Patient characteristics and time delays to treatment southwest Ethiopia January to December 2015

Variable		Total delay(days)			Total
		> 30 (Exposed) *N* = 574	<=30(non exposed) *N* = 161		
		n(%)	n(%)	*P* value	n(%)
Type of TB	Pulmonary positive	277(48.3)	96(59.6)	0.02	373(50.7)
	Pulmonary negative	167(29.1)	46(28.6)		213(29.0)
	Extra pulmonary	130(22.6)	19(11.8)		149(20.3)
HIV status	Positive	57(9.9)	11(6.8)	0.4	68(9.3)
	Negative	517(90.1)	150(93.2)		667(90.7)
Mode of TB diagnosis	Bacteriological	277(48.3)	96(59.6)	0.04	373(50.6)
	Clinical	297(51.7)	65(40.4)		363(49.4)
Treatment center	Hospital	206(35.9)	60(37.3)	0.02	266(36.2)
	Health center	368(64.1)	101(62.7)		469(63.8)
Action before HCF^a^ visit	None	440(76.7)	146(90.7)	0.04	586(79.7)
	Took actions^b^	134(23.3)	15(9.3)		149(20.3)
Place TB diagnosis made	Public	493(85.9)	143(88.8)	0.09	636(86.5)
	Private	81(14.1)	18(11.2)		99(13.5)
Hospitalized for TB illness	Yes	15(2.6)	4(2.5)	0.5	19(2.6)
Knowledge about TB	Poor	159(27.7)	31(19.3)	0.8	190(25.8)
	Good	415(72.3)	130(80.7)		545(74.2)
Patient delay	Median(IQR)days	30(16–57)	14(10–17)	< 0.001	25(15–36)
Provider delay	Median (IQR)days	31(16–64)	7(3–10)	< 0.001	22(9–48)
Pre-diagnosis cost	Median(IQR) US$^c^	119.1(74.0–221.6)	48.2(36.3–66.8)	< 0.001	
Post-diagnosis cost	Median(IQR) US$^c^	93.7(56.9–141.3)	78.8(41.5–113.7)	< 0.001	
Treatment information adequate	Yes	279(48.6)	78(48.4)	0.04	357(48.6)
	No	295(51.4)	83(51.6)		378(51.4)
Initial weight	Mean(±SD) Kg	49.0(8.6)	47.7(8.5)	0.9	48.7(± 8.6)

^a^Healthcare Facility, ^b^self treatment, traditional healer, holy water, ^c^(1US$ = 20.56Birr)

### Follow-up and treatment outcomes

After initiation of treatment, patients had undergone weight and sputum smear monitoring as per the recommended schedules. Accordingly, 501(68.2%), 266(36.2%) and 239(32.5%) of the cases had documented weight at end of second, fifth and sixth months of treatment respectively. Thus, a statistically significant difference in mean weights were observed between baseline and end of second month (t _df = 500_ = 13.94, *p* < 0.001), between sixth month and baseline weight (t _238_ = 11.81, *P* < 0.001). On the other hand, among those smear positive pulmonary TB cases (373(50.7%)) eligible for monitoring of sputum smear, 231(61.9%), 200(53.6%) and 178(47.5%) had documented sputum smear result at end of second, fifth and sixth months of treatment respectively. So, among those with documented follow-up sputum result, 225 (97.4%) from both groups converted to negatives at the end of second month treatment *p* = 0.5) (Table 3).

**Table 3:** Follow-up characteristics and outcomes across delays to treatment southwest Ethiopia January 2015 to june 2016

Variable		Total delay(days)			
		> 30(exposed) *N* = 574	<=30 (non exposed) *N* = 161		Total
		n(%)	n(%)	*P* value	n(%)
Sputum smear end of 2^nd^month(*n* = 373)	Positive	3(1.1)	3(3.0)	0.5	6(1.6)
	Negative	158(57.9)	67(67.0)		225(60.3)
	Not available	112(41.0)	30(30.0)		142(38.1)
Sputum smear end of 5th month(*n* = 373)	Positive	4(1.5)	1(1.0)	0.4	5(1.3)
	Negative	137(50.1)	58(58.0)		195(52.3)
	Not available	132(48.4)	41(41.0)		173(46.4)
Sputum smear end of 6th month(*n* = 373)	Negative	127(46.5)	51(51.0)	0.3	178(47.7)
	Not available	146(53.5)	49(49)		195(52.3)
Sputum check up after diagnosis(*n* = 373)	None	112 (41.0)	30(30.0)	0.3	142(38.1)
	At least once	161(59.0)	70(70.0)		231(61.9)
Weight end of 2ndmonth	Mean(SD)	51.2(8.6)	50.2(8.6)	0.9	51.3(8.6)
Weight change end of 2^nd^ month	Unchanged/lost	93(16.2)	30(18.6)	0.2	123(16.7)
	Gained	298(52.0)	80(49.7)		378(51.5)
	Unknown	183(31.8)	51(31.7)		234(31.8)
Weight end of 5^th^month	Mean(SD)	54.2(8.7)	51.8(9.5)	0.5	53.6(8.9)
Weight end of 6th month	Mean(SD)	54.2(10.3)	52.9(9.0)	0.1	53.9(9.9)
Treatment out come	Cured	126(21.9)	51(31.7)	0.04	177(24.2)
	Treatment complete	354(61.5)	96(59.6)		450(61.1)
	Loss to follow-up	33(5.7)	5(3.1)		38(5.2)
	Died	26(4.5)	3(1.9)		29(3.9)
	Treatment failure	4(0.7)	1(0.6)		5(0.7)
	Not evaluated^a^	31(5.4)	5(3.1)		36(4.9)
Treatment success (*n* = 699)	Unsuccessful	63(11.6)	9(5.8)	0.01	72(10.3)
	Successful	480(88.4)	147(94.2)		627(89.7)

^a^cases transferred out to other treatment centers

At the end of follow-up, 699(95.1%) TB patients had documented treatment outcome of whom 627(89.7%, 95%CI:87.2–91.7%) had successfully completed their treatment. However, among those smear positive TB cases, only 177(47.5%) were declared cured. The treatment success is significantly different among cases initiated treatment beyond and within 30 days of onset of illness (88.4% vs 94.2%, *P* = 0.01), respectively. Thus, significant difference in proportions of death (4.8% Vs 1.9%), treatment failure (0.7% Vs 0.6%) and loss to follow-up (6.1% vs. 3.2%), respectively, among those initiated treatments beyond and within 30 days of onset *p* = 0.04. The treatment success across HIV positive and negatives respectively was 75.8% vs 91.1%, *p* < 0.001. Disaggregation of the treatment success among bacteriologicaly confirmed (smear positive pulmonary) and clinically diagnosed cases respectively revealed 90.1% vs 89.3%, *p* = 0.7. Furthermore, we found no statistically significant differences in treatment success among smear positive pulmonary (90.1%), smear negative pulmonary (88.0%) and extra pulmonary cases (91.1%) (*p* = 0.6).

### Predictors of unsuccessful treatment outcomes

Treatment outcome of the study patients varied significantly across time elapsed for initiation of treatment. Patients delayed for > 30 days to initiate treatment had more than twice higher risk of having unsuccessful outcome Adjusted relative risk [(ARR) = 2.02, 95% confidence interval (CI); 1.03,3.95). Moreover, being older than 65 years, (ARR = 3.83,95% CI; 2.04,6.1), HIV co infection (ARR = 1.93,95% CI; 1.23,3.01) and treatment center being hospital (ARR = 3.78, 95% CI;2.25,6.36) independently predicted higher risk of unsuccessful outcomes. On the other hand, weight gain at the end of second month treatment (ARR = 0.45,95% CI;0.22,0.91) predicted lower risk of unsuccessful outcome. Those patients gained weight at the end of two-month treatment compared to the baseline had about 60% lower risk of having unsuccessful outcome (Table 4).

**Table 4:** Predictors of TB treatment outcome in districts of southwest Ethiopia January 2015 to June 2016

Variable		Treatment success		Crude Relative Risk (CRR) (95%CI)	Adjusted relative risk (ARR) (95% CI)
		Unsuccessful n(%)	Successful n(%)		
Gender	Male	44(10.3)	382(89.7)	Ref.	Ref.
	Female	28(10.3)	245(89.7)	0.9(0.63,1.56)	0.87(0.61,1.25)
Age group(years)	18–34	48(10.0)	433(90.0)	Ref.	Ref.
	35–65	20(9.9)	183(90.1)	0.9(0.60,.62)	0.88(0.68,1.12)
	> 65	4(26.7)	11(73.3)	2.67(1.11,6.45)	3.82(2.4,6.10)*
Educational status	Illiterate	25(12.3)	178(87.7)	Ref.	Ref.
	Completed primary	37(9.9)	338(90.1)	0.8(0.50,1.29)	0.93(0.61,1.41)
	Secondary & above	10(8.3)	111(91.7	0.67(0.33,1.35)	1.00(0.64,1.56)
Treatment center	Hospital	31(12.6)	215(87.4)	1.39(0.9,2.16)	3.78(2.4,6.35)*
	Health center	41(9.1)	412(90.9)	Ref.	Ref.
Action before HCF ^a^visit	None	57(10.3)	498(89.7)	Ref.	Ref.
	Took action	15(10.4)	129(89.6)	1.01(0.59,1.74)	0.81(0.50,1.30)
Mode of TB diagnosis	Bacteriological	35(9.9)	320(90.1)	Ref.	Ref.
	Clinical	37(10.7)	308(89.3)	1.09(0.7,1.7)	0.86(0.67,1.10)
HIV status	Positive	15(24.2)	47(75.8)	2.7(1.63,4.5)	1.93(1.23,3.01)*
	Negative	57(8.9)	580(91.1)	Ref.	Ref.
Weight change end of 2nd month	No change/lost	13(11.2)	103(88.8)	Ref.	Ref.
	Gained	15(4.1)	354(95.9)	0.36(0.18,0.74)	0.45(0.22,0.91)
	Unknown	44(20.6)	170(79.4)	1.83(1.03,3.26)	4.34(2.47.7.65)*
Total delay(days)	<=30	9(5.8)	147(94.2)	Ref.	Ref.
	> 30	63(11.6)	480(88.4)	2.01(1.02,3.95)	2.02(1.03,3.95)*
Treatment information provided	Inadequate	39(11.0)	314(89.0)	0.86(0.56,1.34)	1.06(0.70,1.63)
	Adequate	33(9.5)	313(90.5)	Ref.	Ref.

^*^statistically significant at *p* < 0.05 ^a^ Healthcare facility

Subgroup analysis among smear positive pulmonary cases revealed delays to initiate treatment (ARR = 1.56,95%CI;1.46,1.65), HIV co infection (ARR = 3.73,95%CI:1.68,5.97), treatment center being hospital (ARR = 1.89,95%CI:1.04,3.45) and age older than 65 years (ARR = 6.49,95%CI:3.60,11.70) as independent predictors of unsuccessful outcomes. Similarly analysis among clinically diagnosed cases showed delayed treatment, HIV co-infection, treatment center being hospital and older than 65 years independently predicted higher risk of unsuccessful outcome. Weight gain at the end of second month (ARR = 0.16,95%CI: 0.05, 0.53) predicted higher risk of unsuccessful outcome among clinically diagnosed cases and having at least one sputum checkup after diagnosis (ARR = 0.17, 95%CI:0.09, 0.33) predicted higher risk of unsuccessful outcomes among smear positive cases. Analysis of treatment outcomes among HIV negatives showed initiated treatment after 30 days of onset (ARR = 2.52,95%CI:1.55, 4.10), weight gain at the end of second month treatment (ARR = 0.27,95%CI:0.12, 0.64) and treatment center being hospital (ARR = 2.33,95%CI:1.33, 4.09) independently predicted unsuccessful treatment outcome (Additional file 1: Tables S1-S3).

## Discussion

This follow-up study revealed patients elapse too long time (median of 55 days) to initiate anti-TB treatment since onset of the illness. Subsequently, we found statistically significant differences in treatment success (94.2% vs 88.4%) respectively among those who initiated treatment within and beyond 55 days of onset. Those patients initiated anti-TB treatment beyond 30 days of onset had higher risk of unsuccessful outcomes including death, lost to follow up and treatment failure. Patients initiated treatment within and beyond 30 days of onset had undergone significantly diverse healthcare seeking practices, patient and provider delays. However, both groups of patients had no significant differences in sputum smear conversion and weight changes at end of second month treatment. The longer delays to initiate treatment accompanied by higher risk of unsuccessful outcomes depict increased morbidity and mortality to patients and prolonged period of transmission to the community. The finding suggests need for prompt detection and treatment of cases to ensure better outcomes among patients and reduce burden in community.

The higher risk of unsuccessful outcome among those patients delayed to initiate anti-TB treatment had been consistently reported in studies from Ethiopia [30] and elsewhere [15]. The increased risk of unsuccessful outcome among patients delayed treatment initiation could be explained by various factors. First, delayed initiation of treatment had been reported to be associated with severe clinical presentation [14, 31] which predict unsuccessful outcomes [32]. In this study, patients delayed to initiate treatment had relatively higher rate of hospitalization (3% vs 2.1%) that would be proxy measure of severe presentation. Second, a delay to treatment is associated with both prescribed and self-treatment those lead to poor treatment outcome [33–35]. In the current study, the majority (84.7%) of the cases had visited an average of 2.2 HCFs until diagnosis of TB at which time both self and prescribed medicines had been used. Third, delays to treatment often accompanied by higher direct and indirect costs that impoverish households [36, 37] and ultimately lead to poor treatment compliance and outcome [36]. In our study we observed significantly higher median pre diagnosis (US$119.1 vs 48.2, *p* = 0.001)and post diagnosis (US$93.7vs.98.8, *p* < 0.001) costs among those delayed to initiate treatment which could explain the increased risk of unsuccessful outcome.

Consistent with studies in Ethiopia [30, 38] and elsewhere [39–42], the current study revealed that HIV co-morbidity increase risk of unsuccessful outcome. Hence, significantly lower (75.8% vs 91.1%, *p* < 0.001) treatment success with higher deaths (12.9% vs. 3.3%, *p* = 0.002) were observed among HIV co infected compared to negatives. The observed lower treatment success among HIV co infected is far below the targeted 90% success to be met by 2020 [2]. The increased risk of unsuccessful outcome among the HIV co infected could be due to significantly prolonged time to initiate care seeking (median of 29 vs.24 days, *P* = 0.04) among HIV co infected patients. Thus HIV co morbidity had been reported to delay anti-TB treatment initiation [13, 43]that explain the high mortality among HIV co infected TB patients [16, 17]. In addition, HIV co infection independently increases risk of unsuccessful outcome due the complex and overlapping drug interactions and toxicities and TB-associated immune reconstitution inflammatory syndrome [44]. Furthermore the increased risk could also be explained by the low uptake of TB/HIV collaborative interventions (39.7% and 47.1% on ART and CPT, respectively) those proved to bring better outcome [45]and predict worse outcome in their absence [40].

We found patients took anti-TB treatment at hospital had higher risk of unsuccessful outcome compared to those treated at health center. This could be due to significantly higher proportion of delays to initiate anti-TB treatment (55.6% Vs46.3%, *p* = 0.02), HIV co infection (14.3% Vs 6.4%, *p* < 0.001) and hospitalization (5.3% Vs 1.1%, *p* = 0.001) among patients treated at hospitals. Studies reported HIV co infection [39, 40], hospitalization [32] and delays to treatment [15, 30] to predict higher risk of unsuccessful outcomes. Moreover, patients treated at hospitals were more of pulmonary negative (36.5% vs 24.7%, *p* < 0.001) and extra pulmonary (21.8% vs 19.4%, *p* < 0.001) those had been reported to predict unsuccessful outcome [46].

Regular monitoring of TB patients during treatment is among standard of TB care, which is used to assess response to therapy and facilitates treatment completion. Accordingly regular sputum and weight monitoring have been recommended TB patients on treatment [47]. Despite low sensitivity and modest specificity of sputum results at the end of intensive phase to predict failure and relapse [48], sputum conversion to negative among those positive at initiation of treatment had been taken as one of the indicator TB control program performance [47]. In the current study no significant differences was observed in sputum smear conversion at end of second month treatment in both patients initiated treatment within and beyond 30 days of onset. Consistent with studies in Ethiopia [46]and elsewhere [49] smear conversion to negative at the end of second month treatment had lower risk of unsuccessful outcome. In this study, 3/6(50%) of those treatment failure cases had positive smear at the end of second month treatment. The higher risk among those positives could be due to possible poor quality of initial therapy and co morbid conditions that interfere with adherence or response [47]. Similarly, weight gain at the end of second month treatment predicted lower risk of unsuccessful outcomes which is in line with reports from Vietnam [50].

This study has some limitations. First, exposure assessment relied upon patient recall of the onset of illness that might be subjected to recall bias and ultimate misclassification bias. But efforts had been made to minimize the bias through use of local and national event listing. The second limitation was inability to measure treatment adherence that could have effect on treatment outcome. Third, we did not test for drug susceptibility so that we could not associate delay with resistance. Lastly, we studied only new adult TB cases so that the findings will not apply to all TB cases. On the other hand, relatively large sample and geographic coverage, reduced selection bias through consecutive enrolment, being prospective design, direct estimation of risk and use of standard outcome ascertainment could be mentioned as strength of the study. Therefore, the study is valid and applies to new TB cases in similar settings.

## Conclusion

TB patients in the study area elapse too long time to initiate anti-TB treatment. The delayed treatment initiation was associated with higher risk of unsuccessful outcome including death, treatment failure and lost to follow-up. Apart from the delayed treatment, HIV co infection, treatment center being hospital, weight change and sputum conversion at the end of second month treatment independently predicted unsuccessful outcomes. Therefore, promotion of early care seeking within community, improving diagnostic, and case holding efficiencies of HCF and TB/HIV collaborative interventions could enhance the TB treatment success. Time delays to TB diagnosis can be reduced by raising community level awareness on TB suggestive symptoms, involving traditional healer, religious and private healthcare institutions in TB case finding, equipping healthcare facilities with rapid diagnostic tests and building capacities of healthcare providers to suspect and diagnose TB.

## Additional file

Additional file 1: **Table S1.** Predictors of unsuccessful outcomes among new smear positive pulmonary TB cases in districts southwest Ethiopia January 2015 to June 2016 (*n* = 355). Table S2 Predictors of unsuccessful outcomes among new clinically diagnosed TB cases in districts southwest Ethiopia January 2015 to June 2016 (*n* = 344). Table S3 Predictors of unsuccessful outcomes among not HIV coinfected TB cases in districts of southwest Ethiopia January 2015 to June 2016. (DOCX 20 kb)

## Abbreviations

ARR: Adjusted relative risk
ART: Antiretroviral therapy
CI: Confidence Interval
CPT: Cotrimoxazole prophylactic therapy
DOTS: Directly Observed Treatment Short course
HCF: Healthcare Facility
HIV: Human Immunodeficiency Virus
IQR: Inter-quartile Range
SD: Standard deviation
TB: Tuberculosis
US$: United States dollar
