# Supplementary material for: Delays to treatment initiation is associated with tuberculosis treatment outcomes among patients on directly observed treatment short course in Southwest Ethiopia: a follow-up study
Source: BMC Pulm Med. 2018 May 2;18:64. doi: 10.1186/s12890-018-0628-2 (PMC5930812; doi:10.1186/s12890-018-0628-2)
Supplement: Supplementary file 1 — Table S1. Predictors of unsuccessful outcomes among new smear positive pulmonary TB cases in districts southwest Ethiopia January 2015 to June 2016 (n = 355). Table S2 Predictors of unsuccessful outcomes among new clinically diagnosed TB cases in districts southwest Ethiopia January 2015 to June 2016 (n = 344). Table S3 Predictors of unsuccessful outcomes among not HIV coinfected TB cases in districts of southwest Ethiopia January 2015 to June 2016. (DOCX 20 kb) [file 12890_2018_628_MOESM1_ESM.docx]

**Supplementary tables**

Table 1: Predictors of unsuccessful outcomes among new smear positive pulmonary TB cases in districts southwest Ethiopia January 2015 to June 2016 (n=355)

| Variable |  | Treatment outcome | | CRR (95% CI) | ARR(95% CI) |
| --- | --- | --- | --- | --- | --- |
|  |  | Unsuccessful  n(%) | Successful  n(%) |  |  |
| Sputum check up after diagnosis | Positive | 5(83.3) | 1(16.7) |  |  |
|  | Negative | 5(2.2) | 218(97.8) | 0.03(0.01,0.1) | 0.05(0.02,0.13) |
|  | Not available | 25(19.8) | 101(80.2) | 0.24(0.14,0.39) | 0.62(0.61,0.72) |
| HIV result | Positives | 6(25.0) | 18(75.0) | 2.73(1.25,5.95) | 2.68(1.40,5.12)* |
|  | Negatives | 29(8.8) | 301(91.2) |  |  |
| Gender | Male | 19(8.5) | 205(91.5) |  |  |
|  | Female | 16(12.3) | 114(87.7) | 1.46(0.78,2.73) | 1.18(0.65,2.12) |
| Treatment center | Hospital | 13(13.1) | 86(86.9) | 1.51(0.79,2.87) | 1.89(1.04,3.45)* |
|  | Health center | 22(8.6) | 233(91.4) |  |  |
| Age group(years) | 18-34 | 23(9.2) | 228(90.8) |  |  |
|  | 35-65 | 10(10.1) | 89(89.9) | 1.09(0.54,2.21) | 1.44(0.82,2.54) |
|  | >65 | 2(50.0) | 2(50.0) | 5.46(1.90,15.7) | 5.70(3.1,10.5)* |
| Total delay(days) | <=30 | 15(7.8) | 177(92.2) |  |  |
|  | >30 | 20(12.3) | 142(87.7) | 2.18(1.12,4.25) | 1.88(1.11,3.17)* |
| Action taken before HCF** visit | None | 30(10.5) | 257(89.5) |  |  |
|  | Took actions | 5(7.5) | 62(92.5) | 0.70(0.28,1.75) | 0.54(0.22,1.32) |

*statistically significant at p<0.05 ** Healthcare Facility

Table 2: Predictors of unsuccessful outcomes among new clinically diagnosed TB cases in districts southwest Ethiopia January 2015 to June 2016 (n=344)

| Variable |  | Treatment outcome | |  |  |
| --- | --- | --- | --- | --- | --- |
|  |  | Unsuccessful  n(%) | Successful  n(%) | CRR(95% CI) | ARR(95% CI) |
| Weight change end of 2^nd^ month | No change/lost | 9(15.5) | 49(84.5) | Ref. | Ref. |
|  | Gained | 4(2.2) | 179(97.8) | 0.14(0.05,0.44) | 0.16(0.05,0.53)* |
|  | Unknown | 24(23.3) | 79(76.7) | 1.50(0.75,3.01) | 3.21(1.59,6.48)* |
| HIV result | Positive | 9(23.7) | 29(76.3) | 2.67(1.36,5.2) | 1.86(1.16,2.98)* |
|  | Negative | 28(9.1) | 279(90.9) | Ref. | Ref. |
| Gender | Male | 25(12.4) | 177(87.6) | Ref. | Ref. |
|  | Female | 12(8.4) | 131(91.6) | 0.67(0.35,1.29) | 0.65(0.43,1.09) |
| Treatment center | Hospital | 18(12.2) | 129(87.8) | 1.28(0.7,2.36) | 2.78(1.63,4.76)* |
|  | Health center | 19(9.6) | 179(90.4) | Ref. | Ref. |
| Age group(years) | 18-34 | 25(10.9) | 205(89.1) | Ref. | Ref. |
|  | 35-65 | 10(9.6) | 94(90.4) | 0.89(0.45,1.79) | 0.76(0.52,1.13) |
|  | >65 | 2(18.2) | 9(81.8) | 1.67(0.45,6.18) | 4.4(1.64,11.81)* |
| Total delay(days) | <=30 | 11(6.8) | 150(93.2) | Ref. | Ref. |
|  | >30 | 26(14.1) | 158(85.9) | 1.49(0.79,2.80) | 2.22(1.12,4.39)* |
| Action taken before HCF** visit | None | 27(10.1) | 241(89.9) | Ref. | Ref. |
|  | Took actions | 10(13.0) | 67(87.0) | 1.30(0.66,2.57) | 0.83(0.56,1.23) |

*statistically significant at p<0.05 ** Healthcare Facility

Table 3: Predictors of unsuccessful outcomes among not HIV coinfected TB cases in districts of southwest Ethiopia January 2015 to June 2016

|  |  | Treatment outcome | | CRR(95% CI) | ARR(95% CI) |
| --- | --- | --- | --- | --- | --- |
|  |  | Unsuccessful  n(%) | Successful  n(%) |  |  |
| Weight change end of second month | No change/Lost | 11(10.7) | 92(89.3) | Ref. | Ref. |
|  | Gained | 10(2.9) | 329(97.1) | 0.27(0.12,0.63) | 0.27(0.12,0.64)* |
|  | Unknown | 36(18.5) | 159(81.5) | 1.73(0.92,3.25) | 2.97(1.54,5.77)* |
| Gender | Male | 34(8.7) | 356(91.3) | Ref. | Ref. |
|  | Female | 23(9.3) | 224(90.7) | 1.07(0.64,1.77) | 0.88(0.55,1.42) |
| Mode of diagnosis | Bacteriological | 29(8.8) | 301(91.2) | Ref. | Ref. |
|  | Clinical | 28(9.1) | 279(90.9) |  | 0.99(0.63,1.58) |
| Treatment center | Hospital | 19(9.0) | 193(91.0) | 1.02(0.59,1.69) | 2.33(1.33,4.09)* |
|  | Health center | 38(8.9) | 387(91.1) | Ref. | Ref. |
| Age group (years) | 18-34 | 38(8.7) | 401(91.3) | Ref. | Ref. |
|  | 35-65 | 15(8.2) | 169(91.8) | 0.94(0.53,1.67) | 0.92(0.53,1.58) |
|  | >65 | 4(28.6) | 10(71.4) | 3.30(1.37,7.97) | 4.33(2.76,6.77)* |
| Total delay(days) | <=30 | 20(6.2) | 304(93.8) | Ref. | Ref. |
|  | >30 | 37(11.8) | 276(88.2) | 2.21(1.29,3.79) | 2.52(1.55,4.10)* |
| Action taken before HCF** visit | None | 43(8.5) | 461(91.5) | Ref. | Ref. |
|  | Took actions | 14(10.5) | 119(89.5) | 1.23(0.69,2.19) | 0.93(0.55,1.60) |

*statistically significant at p<0.05 ** Healthcare Facility
